# Restoring neuroplasticity after CNS trauma: cell therapy approaches in spinal cord and traumatic brain injury

**DOI:** 10.1186/s12967-026-07933-5

**Published:** 2026-06-12

**Authors:** Ana T. Palha, Marta F. Lima, Melyssa Carvalho, Jonas Campos, Belém Sampaio-Marques, António J. Salgado

**Affiliations:** 1 Life and Health Sciences Research Institute (ICVS), School of Medicine, University of Minho, Campus de Gualtar, Braga, 4710-057 Portugal; 2 ICVS/3B’s Associate Lab, PT Government Associated Lab, Braga/Guimarães, 4806-909 Portugal

**Keywords:** Central nervous system, Spinal cord injury, Traumatic brain injury, Stem cell, Therapy

## Abstract

**Background:**

The central nervous system (CNS) has a limited regenerative capacity, rendering traumatic injuries such as spinal cord injury (SCI) and traumatic brain injury (TBI) highly disabling and difficult to treat. These insults trigger complex pathophysiological cascades, including extensive cell death, sustained inflammation, and the formation of a hostile inhibitory microenvironment that compromises neural plasticity and hampers tissue regeneration. The multifactorial nature of these mechanisms, together with a fragmented understanding of CNS plasticity, has hindered the development of effective therapeutic interventions.

**Main body:**

In recent years, cell-based therapies have emerged as promising strategies to support neural repair and induce pro-regenerative processes. This approach encompasses multiple cell types, including bone marrow–derived mesenchymal stem cells (BMSCs), adipose-derived stem cells (ASCs), umbilical cord mesenchymal stem cells (UCMSCs), as well as neural progenitor cells (NPCs) and olfactory ensheathing cells (OECs). Clinical studies in SCI have reported functional improvements, particularly following treatment with BMSCs and peripheral blood–derived stem cells, although substantial methodological heterogeneity limits definitive conclusions. In TBI, clinical evidence remains more limited; however, preclinical studies consistently demonstrate the neuroprotective and regenerative potential of mesenchymal stem cell–based therapies. Beyond direct cell transplantation, increasing attention has been given to cell-free approaches, including secretome and extracellular vesicle–based strategies, which recapitulate many of the beneficial effects while potentially overcoming safety and logistical constraints.

**Conclusions:**

Overall, both cell-based and cell-free therapeutic strategies show significant potential to enhance neuroplasticity, attenuate secondary injury, and promote functional recovery following CNS trauma. Nevertheless, successful clinical translation will require larger, well-controlled trials and the establishment of standardized protocols addressing optimal timing, dosage, and routes of administration.

## Background

Our current understanding of neuroplasticity responses in central nervous system (CNS) trauma is fragmented. Although recognized as being crucial for the intrinsic capacity of the CNS to re-adapt following injury, the current extent to which plasticity occurs in different traumas is underexplored [1]. Novel therapeutic interventions such as the use of cell therapy and its derivatives have been shown to modulate different plasticity mechanisms [2]. In this work, the current advances in the use of cell therapies for traumatic brain injury and spinal cord injury (TBI and SCI) are revised and discussed, giving special attention to their modulatory role of pathophysiologic mechanisms that can impact plasticity responses.

### Spinal cord injury (SCI)

SCI is defined as a neurological condition that may result in either temporary or permanent impairments [3]. According to the cause of injury, SCI can be divided into traumatic and non-traumatic [4, 5]. Traumatic spinal cord injury (TSCI) occurs when the spinal cord tissue is damaged due to an external physical trauma, whereas non-traumatic spinal cord injury (NTSCI) is caused by diverse etiologies, such as tumors, infections and degenerative diseases [6]. The incidence of TSCI is notably higher that NTSCI, consequently, the majority of the studies has predominantly focused on TSCI [4, 7].

Among cases of TSCI, the most common causes of injury are traffic accidents, followed by falls, acts of violence, and sports-related injuries [4, 7]. The incidence of TSCI is influenced by both age and sex, with higher prevalence among younger males compared to females, however this ratio tends to decrease with advancing age. Nevertheless, among all age groups, TSCI are more common in males than in females [7, 8].

SCI can be classified as either complete or an incomplete injury, depending on the degree of neurological impairment (Table 1) [9]. A complete SCI is defined as the total loss of voluntary motor control and sensory function below level of the injury. In contrast, in an incomplete SCI, some functions are preserved below the injury site [10]. However, this definition is inadequate in certain clinical scenarios. To address these discrepancies, the American Spinal Injury Association (ASIA) created a universal standardized classification system. This scale categorizes injuries from grade A to grade E. Grade A represents a complete SCI, while grade E indicates normal motor and sensory functions (Table 1) [10, 11].

**Table 1:** ASIA scale. Definition of ASIA grade in the context of spinal cord injury according to standard ASIA/ISNCSCI terminology [12–14]

Grade	Definition
A	Complete injury. There is no preserved sensory or motor function in the sacral segment (S4–S5).
B	Sensory incomplete. Motor function is preserved below the injury, as is sensory function, which includes the sacral segments S4 and S5 (i.e. light touch or a pinprick at S4 and S5, or deep anal touch).
C	The motor impairment is classified as incomplete. Motor function is retained at the most caudal segment, evidenced by voluntary anal contraction (VAC), or the individual fulfils the criteria for sensory-incomplete status, with motor function preserved in more than three segments below the ipsilateral motor level on at least one side of the body.
D	Motor incomplete. Motor-incomplete status is characterized by the preservation of at least 50% of key muscle functions below the level of injury with a muscle grade ≥ 3.
E	Normal. Should the patient exhibit normal results in the sensory and motor function tests conducted using the ISNCSI, and in the event that prior deficits are documented.

**Fig. 1:**
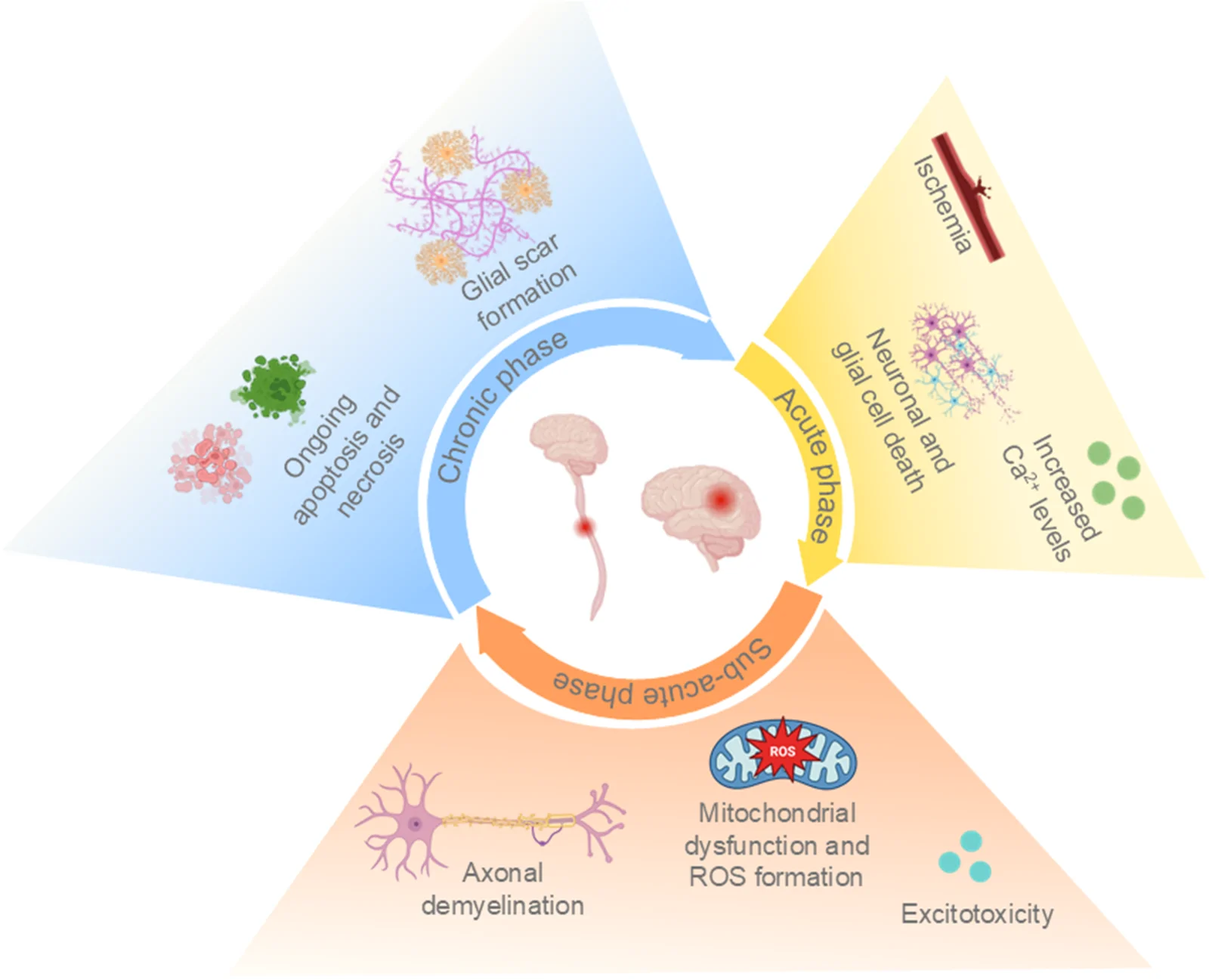
Pathophysiological mechanisms of secondary injury underlying Spinal Cord Injury (SCI) and Traumatic Brain Injury (TBI). Schematic representation of the main pathophysiological events in secondary injury, including ischemia, blood–CNS barrier disruption, ionic imbalance, and excitotoxicity (acute phase); oxidative stress, mitochondrial dysfunction, and axonal demyelination (sub-acute phase); cellular death and glial scar formation (chronic phase). These processes collectively impair plasticity and regeneration. Ilustration created with Bio Render (https://www.biorender.com/)

**Fig. 2:**
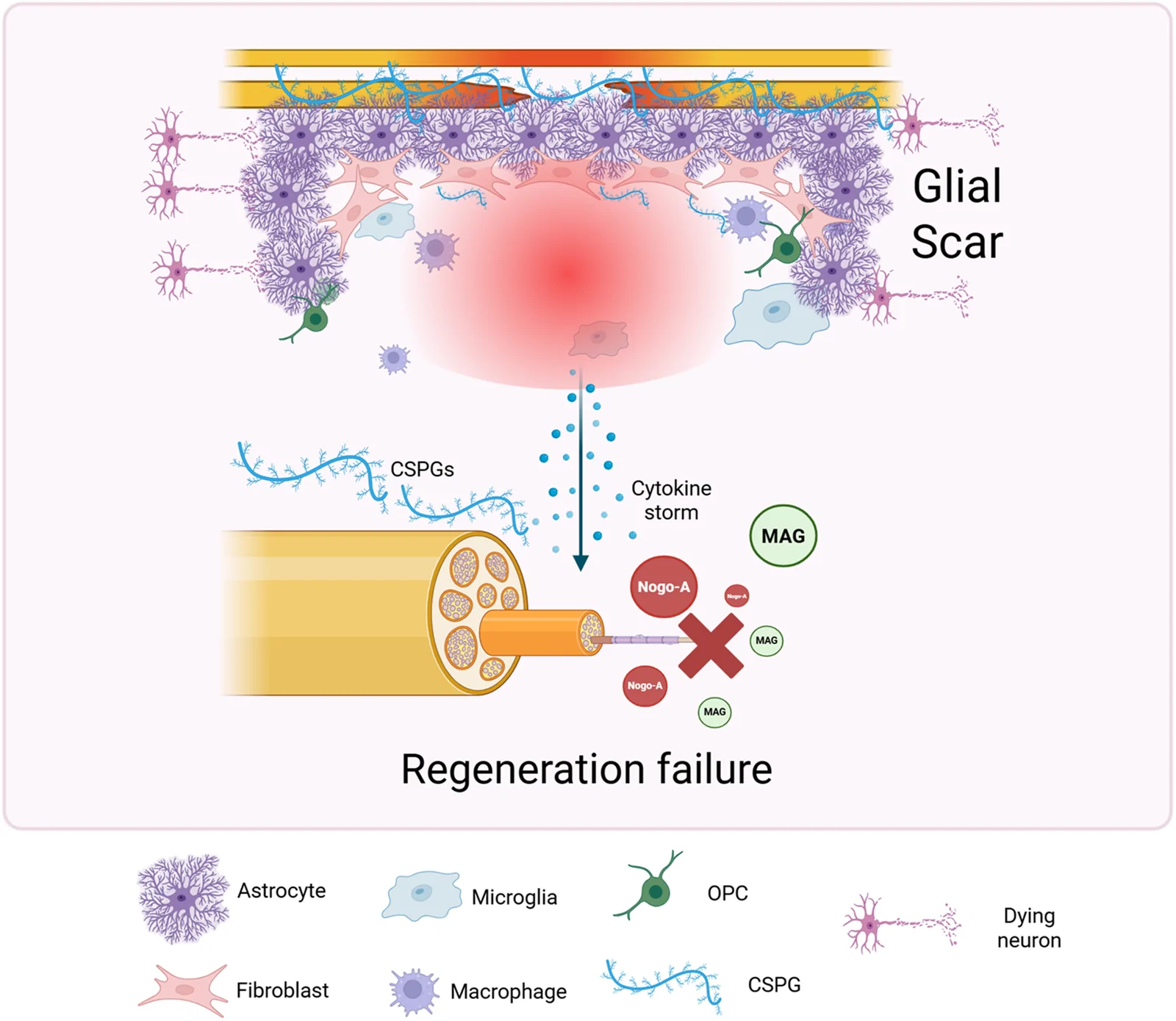
Regeneration barriers following CNS trauma. Illustration of the inhibitory environment established in a subacute to chronic phase after CNS injury. It represents the formation of a fibroglial scar, composed by reactive astrocytes, NG2 glia, fibroblasts and activated immune cells, posing a physical and biochemical barrier. The secretion of major molecular (inflammatory cytokines) and cellular components (CSPGS, MAG, Nogo-A, among others) further induces the hostile environment for axon growth and plasticity. Illustration created with Bio Render (https://www.biorender.com/)

The pathophysiology of SCI is a complex and dynamic process, characterized by two temporally connected phases: primary and secondary injuries [3, 5, 15]. Primary injury is characterised by direct physical damage to the spinal cord, typically resulting from fractures or vertebral dislocation in cases of TSCI. In NTSCI, primary injury most commonly arises from inflammatory processes, tumours, or vascular events [6]. Initially, these insults lead to hemorrhage, as well as disruption of glial membrane and axonal network [3, 5, 15]. This is followed by the secondary injury response that encompasses a cascade of biochemical and cellular events. This response can be divided in three phase. The first phase, known as the acute phase, comprises the spinal ischemia, resulting from reduced blood flow and alterations in blood-spinal cord barrier permeability that leads to edema formation and allow peripheral immune cells to infiltrate. During this phase, an ionic imbalance occurs, which exacerbates neuronal and glial cell death, leading to elevated intracellular calcium levels. In addition to calcium overload, excessive glutamate is released into the extracellular space, triggering excitotoxicity which contributes to further cellular damage. During the sub-acute phase, persistent excitotoxicity disrupts normal mitochondrial function, resulting in the accumulation of reactive oxygen species (ROS). This oxidative stress leads to axonal demyelination and neuronal loss at the injury site. Furthermore, the sustained disruption of the blood-spinal cord barrier exacerbates neuroinflammation, all of which contribute to the creation of a more hostile environment. These events are followed by the chronic phase, which can persist for several years in patients with SCI. This phase is characterized by ongoing apoptosis, necrosis, axonal degeneration and demyelination. Ultimately, these pathological processes lead to the development of a cystic cavity and the maturation of a glial scar (Fig. 1) [3, 5, 15]. This last event constitutes a significant barrier to robust plasticity as it limits axonal growth through the injury epicenter (Fig. 2) [5].

### Traumatic brain injury (TBI)

Traumatic brain injury (TBI) is defined as a nondegenerative, noncongenital insult to the brain caused by an external force, resulting in temporary or permanent impairments in cognitive, physical and psychological functions [16].

The most common causes of TBI are road traffic accidents and falls, which together account for more than half of all cases. The most affected group with TBI are young males. However, older adults also represent a significant proportion of cases due to domestic falls. Despite these age-related variations, TBI is associated with high rates of mortality and disability, making it a major public health concern [17, 18].

According to the affected brain regions, TBI is intimately linked to cognitive and behavioral alterations, as well as to chronic encephalopathies [19]. The resulting impairments also depend on the type of physical impact, characterized into closed-head (compression from blunt impact), penetrating (laceration from penetration) and explosive blast (widespread injury from shock waves), all culminating in periods of unconsciousness and brain tissue damage [20]. All types of impact eventually result in focal localized contusions or diffuse injury to other brain regions, with different grades of severity [21].

Several criteria are currently implemented to classify TBI severity as mild, moderate or severe, although not elucidating the responsible pathophysiologic mechanisms [22]. These are a combination of Glasglow Coma Scale (GCS), duration of loss of consciousness, post-traumatic amnesia and structural imaging of the damaged tissues, useful for clinical management [19].

The Glasgow Coma Scale (GCS) was developed as a standardized evaluation tool [23], that evaluate three clinical parameters: eye opening, verbal response and motor response, with a total score ranging from 3 to 15. Each parameter is scored based on predefined criteria, and the sum of these scores is then used to assess the severity of the TBI. A mild injury (GCS score 13–15) is associated with structural imaging mostly normal and symptoms of headache, nausea and slowed cognition. In moderated TBI (GCS score 9–12), there may be a visible anatomical damage, a prolonged loss of consciousness and more pronounced cognitive and coordination impairments. Lastly, a severe injury (GCS score 3–8) present significant abnormal anatomical damage and may include coma and post-traumatic amnesia prolonged or permanent, having high rates of mortality and severe disability [24, 25].

In general, a GCS score of 13–15 indicates a mild TBI, representing the lowest level of severity. Scores ranging from 9 to 12 corresponds to a moderate TBI, and a score of 3–8 reflects a severe TBI, which is typically associated with coma or markedly reduced responsiveness [26–28]. However, the GCS has limitations to accurately characterize specific TBI scenarios. These problems arise from factors such as patient health status, scoring ambiguity, and limited long-term outcome prognosis value [27, 28].

Nonetheless, similar pathoanatomic features of brain trauma usually share common pathophysiologic mechanisms, being difficult to link physical injury to specific and targetable molecular events [29].

The pathophysiology of TBI can be divided into two sequential stages: primary and secondary injury. The primary injury is the moment at which physical trauma causes irreversible damage to brain tissue, compromising the blood-brain barrier function by increasing its permeability. In this stage, if the skull is open the primary injury is classified as penetrating (open-head), if the skull remains intact, the lesion is referred as nonpenetrating (closed-head or blunt). The subsequent pathological processes are categorized as secondary injury, which involves a complex cascade of biochemical, metabolic, and cellular events that exacerbate the initial damage. These secondary alterations are not limited to the site of the primary lesion and can contribute to widespread neurological dysfunction. Similar to the SCI, the secondary phase of TBI involves a range of pathological mechanisms, including ischemia, excitotoxicity, inflammation, mitochondrial dysfunction and massive cell death, which leads to neurological dysfunction. In a more chronic stage, these processes may lead to glial scar formation, further impairing neural recovery and function (Fig. 1) [30–33].

## The failure of CNS regeneration

The potential for functional recovery in patients with SCI and TBI largely depends on the type and severity of the injury [5, 9]. However, sufficient tissue recovery in human CNS injuries is hindered by a limited capacity for neuronal regeneration. This limited regenerative drive is due to both intrinsic and extrinsic factors (Fig. 2) [1, 34, 35].

CNS has a limited intrinsic ability to regenerate neurons and remyelinate after injury, primarily due to the genetic landscape of post-mitotic neurons and the extracellular environment established following the lesion [34–36].

At the cellular level, neuronal intrinsic mechanisms hampers plasticity and successful regeneration attempts due to a repressesed activity of regeneration associated genetic programs that limits axonogenic and neuritogenic potential [37–40].

For instance, increased activity of phosphatase and tensin homolog deleted on chromosome 10 (PTEN) is causally linked to reduced axonogenic potential and regeneration across the injury site [40]. This intrinsic alterations inhibits keys mechanisms responsible for cell survival and axonal growth, such as Phosphatidylinositol 3-kinase (PI3K)/Protein Kinase B (Akt) and mTOR pathways which are crucial for survival and neurite extension [41–44]. In parallel, white matter repair is further inhibited by the activity of molecules such as the Nogo-A protein and myelin-associated glycoprotein (MAG) [45, 46]. These molecular signals activate transcriptional programs that further suppress neurite outgrowth. This occurs through the activation of Rho, which in turn stimulates Rho-associated kinase (ROCK). Rock then leads to cytoskeletal rearrangements of actin and microtubules. Consequently, growth cones retract, which further inhibits neurite outgrowth [47–49]. In addition to these extrinsic mechanisms, one of the most prominent extrinsic barriers to regeneration after SCI and TBI is the formation of a glial scar. This scar develops through the deposition of connective tissue and the excessive production of chondroitin sulfate proteoglycans (CSPGs) [34, 35]. The glial scar inhibits axonal regrowth at the lesion site, a process that is essential for neuroplasticity and the establishment of new neuronal connections that could potentially compensate for the sustained damage [5, 34, 35]. However, the glial scar does not only present negative outcomes. This barrier, which is mainly composed of reactive astrocytes, plays an important role in wound containment, limiting inflammation, and preserving tissue integrity after injury. Furthermore, some experimental studies suggest that these reactive astrocytes facilitate and even support axonal regeneration when combined with appropriate growth factors. They act as a scaffold that can bridge the lesion site and allow axon extension across the injury. Therefore, ablating of the glial scar does not necessarily create a suitable environment for axon regrowth [50, 51].

Given these intrinsic and extrinsic factors, the natural capacity of CNS for neuroplasticity, which is defined as its ability to reorganize and adapt in response to stimuli is profoundly affected. The pathophysiological processes underlying SCI and TBI are highly complex and, as stated above, further compromise neuroplasticity (Fig. 2) [5, 34–36]. To overcome these barriers, increasing focus has been directed towards stem cell-based therapies to treat spinal cord and traumatic brain injuries [52]. These therapeutic approaches leverage cell-based therapies not only to replace damaged tissue but also to secrete neuroprotective and anti-inflammatory factors. Collectively, these properties support regeneration and promote functional recovery of the CNS following injury [53–56].

## Preclinical results, exploring mechanisms of plasticity and regeneration

### Spinal cord injury (SCI)

**Fig. 3:**
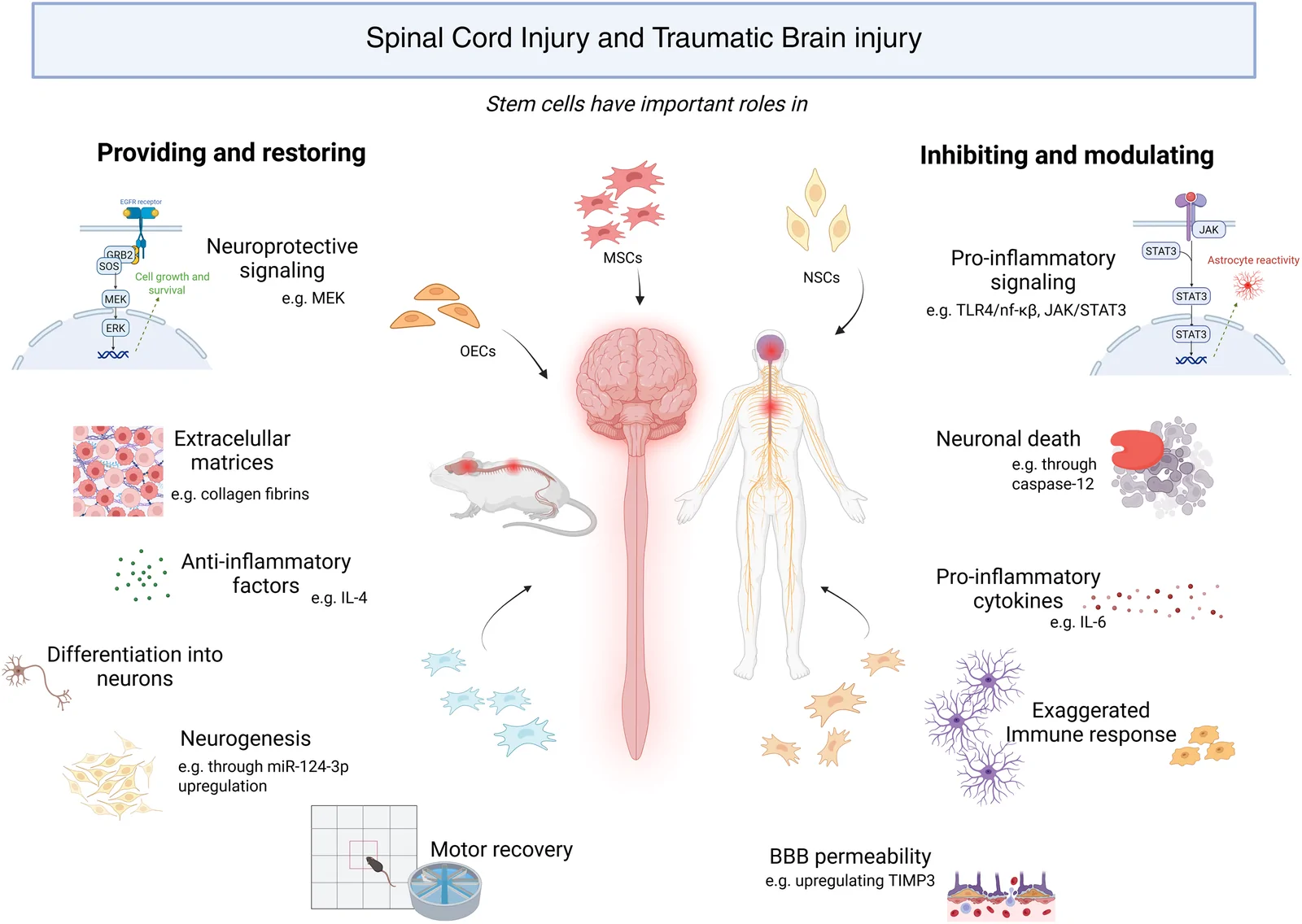
Schematic illustration of the roles of stem cells in SCI and TBI. Stem cells contribute to providing and restoring functions by promoting neuroprotective signaling, producing extracellular matrices, releasing anti-inflammatory factors, differentiating into neurons, stimulating neurogenesis, and supporting motor recovery. They also aid in inhibiting and modulating damage by reducing pro-inflammatory signaling, limiting neuronal death, decreasing pro-inflammatory cytokines, dampening exaggerated immune responses, and regulating BBB permeability. OECs: olfactory ensheathing cells; MSCs: mesenchymal stem cells; NSCs: neural stem cells. Ilustration created with Bio Render (https://www.biorender.com/)

Preclinical SCI studies commonly employ rodent models—particularly rats and mice—to investigate potential therapeutic strategies [57], including the role of stem cells in promoting neural repair and functional recovery (Fig. 3). Injury paradigms include compression, contusion, transection, and hemisection [57, 58], which allow for interrogation of diverse injury phenotypes and pathophysiological responses. Functional recovery is typically assessed using the Basso, Beattie, and Bresnahan (BBB), and its mouse analogous Basso mouse scale (BMS), locomotor scale, standardized tool for evaluating hindlimb motor function [59, 60].

Bone Marrow–Derived Mesenchymal Stem Cells (BMSCs) support neuroprotection and improvement of locomotor function by downregulating pro-inflammatory signaling pathways such as Toll-Like Receptor 4 (TLR4) and Nuclear Factor kappa-light-chain-enhancer of activated B cells (NF-κB) and reducing caspase-12–mediated stress responses and neuronal death [61, 62]. BMSCs also provide extracellular matrices such as collagen fibrils that support Schwann cell-associated axon growth and reduce glial cells and cavity formation [62]. Additionally, BMSCs promote blood vessel formation around the injury site, which is essential for nutrient supply in SCI [63].

Peripheral-blood mononuclear cells (PBMSCs), derived from sources like human umbilical cord blood or bone marrow, promote spinal cord repair by restoring neuroprotective Neurotrophin-3 (NT3) and Mitogen-activated protein Kinase-1 (MEK-1) signaling [64]. Their transplantation enhances 5-HT fiber growth, oligogenesis, and angiogenesis, while reducing astrogliosis and cavity formation, leading to improved functional recovery [64–66]. These effects are partly mediated by early secretion of soluble factors and immunomodulation [65].

Transplantation of Umbilical Cord–Derived Mesenchymal Stem Cells (UCMSCs) accelerated motor recovery and increased pain thresholds, while reducing ED1-positive microglia/macrophages, cavity size, and proinflammatory cytokines (Interleukin-6 (IL-6), Interleukin-7 (IL-7), Interferon-gamma (IFN-γ), Tumor Necrosis Factor-alpha (TNF-α)), and increasing anti-inflammatory and neurotrophic factors (Interleukin-4 (IL-4), Interleukin-13 (IL-13), Brain-Derived Neurotrophic Factor (BDNF), Glial cell line-Derived Neurotrophic Factor (GDNF) [67–70]. It also decreased Glial Fibrillary Acidic Protein (GFAP) and neuronal apoptosis, likely via downregulation of p38 Mitogen-Activated Protein Kinase (p38 MAPK) [71], and enhanced neurogenesis through the microRNA miR-124-3p upregulation, promoting neuronal differentiation and axon growth [68].

Adipose-Derived Stem Cells (ASCs) improve outcomes by inhibiting microglial activation and modulating inflammatory pathways, including Jagged1 ligand/Notch receptor (Jagged1/Notch) and Janus kinase / Signal Transducer and Activator of Transcription 3 (JAK/STAT3) [72], while promote neuronal survival by upregulating Ciliary Neurotrophic Factor (CNTF) expression, reducing apoptosis via JAK/STAT signaling [73] and by upregulating progranulin, a neuroprotective glycoprotein that acts as a negative regulator of inflammation [74]. After transplantation of ASCs combined with a fibrin matrix, mRNA levels of Platelet-Derived Growth Factor Receptor (PDGFR) and Heat Shock Protein Family A Member 1B (HSPA1b) were significantly upregulated, indicating apoptosis inhibition and enhanced support of vascular growth, cell proliferation, and matrix remodeling [75].

Neural Stem Cells (NSCs) contribute to repair by differentiating into neurons that extend long-distance axons, form synapses, and promote recovery through mechanistic Target of Rapamycin (mTOR)-dependent mechanisms [76]. In addition to forming synaptic relays, NSCs secrete trophic factors such as Nerve Growth Factor (NGF), BDNF, and GDNF that support host axon growth and survival [77]. Neural Progenitor Cells (NPCs) enable corticospinal tract regeneration by bridging lesion sites and attenuating glial scarring [78]. In primates, those cells differentiated into all three neural lineages and reduced lesion cavity size [79].

Olfactory Ensheathing Cells (OECs), derived from the olfactory mucosa and bulb, also enhance recovery and axonal support by secreting neurotrophic factors like NGF, BDNF and GDNF, and promoting NSCs differentiation into neurons [80]. Their ability to guide early, faster and shorter immune responses via Interleukin-1 beta (IL-1β) upregulation supports the modulation of the injury environment and long-term reduction of astrogliosis [81–83] also contributing to remyelination of spared axons [81].

In summary, multiple cell types show promise for SCI repair through neuroprotection, inflammation modulation, and repair. Combinatorial strategies with these cells may further enhance spinal cord plasticity and regeneration [80, 84–87], underscoring the central role of cell-based therapies as a promising avenue for SCI repair.

### Traumatic brain injury (TBI)

Stem cell therapies for TBI have been exploited in plentiful pre-clinical models, where various cell sources have been tested, using different delivery routes of administration and injury paradigms, highlighting their diverse roles in repair and recovery (Fig. 3).

Therapeutic outcomes are generally investigated by assessing sensorimotor and cognitive functions, through coordination assessments, hindlimb function and neurological scoring tests, namely Morris Water Maze, modified Neurological Severity Score and rotarod [88, 89]. Anatomical damage is also used as a predictive measure of therapeutic efficacy through lesion volume and tissue damage [88, 89]. Direct brain delivery (into the cavity or intracranial) is the most employed method for cell transplantation, whereas systemic and intranasal strategies offer a less invasive alternative for clinical context [88].

The efficacy and safety of Mesenchymal Stem Cells (MSCs) have been considerably assessed in the mentioned pre-clinical settings, supporting their current transition into clinical trials, and unveiling multiple mechanisms behind their therapeutic effects. Bone marrow MSCs [90] are the most employed, followed by cells from umbilical cord [91], adipose tissue [92] and placenta [88, 93]. The MSCs-sources have shown overall improvements in sensorimotor and cognitive functions and in anatomical damage, being intimately associated with the delivery route and timing of administration [94, 95]. It is now well established that their reported therapeutic potential is mostly attributed to an intrinsic paracrine activity in situ. They secrete a cocktail of factors enriched in immunomodulatory cytokines, neurotrophic and angiogenic factors, which collectively promote neurogenesis and cell survival pathways, while modulating cell death mechanisms [96, 97] (Fig. 3). They present homing properties, by migrating to injured and inflamed tissues through mechanisms involving adhesion molecules such as Vascular cell adhesion protein 1 (VCAM-1) [98]. Moreover, secretion of trophic factors, such as BDNF and Insulin-Like Growth Factor 1 (IGF-1) induces the recruitment of endogenous progenitor cells, promoting tissue repair and replacement of lost cells [97, 99]. Interestingly, contusion volume preservation and reduced anatomical damage results have been reported to correlate with later post-treatment evaluation time-points [90, 100]. This may reflect the principal mechanism of action of MSCs, which involves an injury microenvironment reprogramming - attenuating detrimental pathways, while inducing a restorative effect of neuroprotection and repair overtime [101, 102]. Additionally, these effects are ample in acute phase administration (within the first week port-injury), complying with the therapeutic potential in attenuating and limiting secondary injury events [88]. For instance, MSCs have been shown to upregulate the expression of metellaproteases inhibitors (TIMP3), contributing to a decreased blood-brain barrier permeability in mouse models of TBI [103]. Their transplantation has been associated with reduced areas of hypoperfusion in brain regions adjacent to the injury site, correlating to decreased cerebral atrophy and improvements in functional recovery [90]. A reduction of pro-inflammatory glial phenotypes and neuroinflammation has also been reported, suggesting that this approach could potentially bridge the gap between immunomodulatory and neuroprotective agents [104, 105].

## Clinical studies

### Spinal cord injury (SCI)

A wide variety of stem cell types, including bone marrow–derived mesenchymal stem cells (BMSCs), hematopoietic stem cells (HSCs), UCMSCs, ASCs, NSCs, NPCs, and OECs, have been tested in clinical trials of SCI. Table 2 highlights trials with rates of ASIA improvement larger or equal to two grades, based on the meta analysis of Ribeiro et al., 2023 [106] and trials that have been published since, focusing on those reporting at least one patient improving two or more grades in the ASIA score. Moving up by two or more ASIA grades represents a clinically meaningful recovery — for example, a transition from motor complete (ASIA A/B) to motor incomplete (ASIA C/D) can profoundly change independence, rehabilitation potential, and quality of life [107].

BMSCs currently provide the most robust clinical evidence base due to the high volume of published trials and consistent functional improvements across diverse patient subgroups, although the greatest improvement was reported by Bryukhovetskiy [108] with autologous peripheral blood stem cells leading to recovery from ASIA A to D. Across the studies summarized in this review, stem cells were predominantly derived from autologous sources. Allogeneic transplantations were less frequent among trials reporting high-grade ASIA improvement studies and were implemented mainly with UCMSCs and NSCs/NPSCs. While allogeneic approaches offer advantages in terms of cell availability and standardization, they may introduce variability related to host immune responses, which could influence engraftment efficiency and functional outcomes [109]. In contrast, autologous cell therapies are generally considered immunologically safer, as they minimize the risk of immune rejection [110]; however, their clinical use is often constrained by the need for cell harvesting, expansion, and processing, making them less suitable for immediate intervention compared to biobanked allogeneic products that can be administered without delay [109]. These differences in cell origin therefore represent an important factor when interpreting treatment efficacy across trials.

Intrathecal (via lumbar puncture) administration was highly frequent and feasible route for all types of stem cells, followed by intralesional injection, while implantation was more frequent when using scaffolds and autografts like Olfactory Mucosal Autograft (OMA). Several successful trials utilized multiple routes simultaneously and at different time points, to maximize cell distribution. While dosages vary widely and the “optimal” dose is still under investigation, in these clinical trials the number of cells delivered ranged from 10^5^ to 10^8^ cells. Finally, while acute treatment is prioritized in preclinical studies, clinical evidence shows a broad therapeutic window extending into the chronic phase (months to years), with patients being treated anywhere from 7 days to 30 years’ post-injury.

It is important to note that there are a considerable number of phase 1 or early-phase clinical trials with small sample sizes and limited controls. For this reason, we emphasize those with the largest observed effect sizes, while acknowledging that these are not representative of the entire body of evidence.

Another important aspect to consider is that the benefits observed in preclinical studies are largely associated with acute interventions, when secondary injury mechanisms are still evolving [111]. Targeting these early pathological processes may allow stem cell–based therapies to modulate the broader secondary injury cascade, acting as a neuroprotective intervention [112]. In contrast, early intervention in clinical settings is frequently limited by practical and logistical constraints, including medical instability in polytrauma patients, pre-existing comorbidities, delays related to patient transfer to specialized centers and access to diagnostic investigations [113]. As a result, many patients receive treatment during the subacute or chronic phases. This discrepancy in treatment timing likely contributes to the more robust efficacy observed in preclinical models compared with the more variable outcomes reported in clinical studies.

When interpreting these stem cell results, it is useful to contrast them with better-studied interventions. According to randomized controlled trials and controlled observational studies, high-dose methylprednisolone sodium succinate (MPS) does not significantly improve long-term motor score recovery or the likelihood of improving by at least one ASIA grade compared to placebo or no treatment [114]. Similarly, in human testing of anti-Nogo-A antibodies, the phase 2b NG101 trial did not improve upper extremity motor scores in acute cervical SCI patients [115], and earlier phase I data showed that of 19 evaluable tetraplegic patients treated with anti-Nogo-A, 2 converted from ASIA A to C [116] but these were small, uncontrolled cohorts.

Taken together, stem cell interventions appear to yield higher proportions of ≥ 2-grade AIS improvements. While results are promising, further studies with larger sample sizes and combination strategies are needed to optimize therapeutic outcomes.

**Table 2:** Scientific publications reporting clinical studies on cell-based therapies in SCI patients achieving a ≥ 2-grade improvement on the ASIA scale, as summarized in the meta-analysis by Ribeiro et al. (2023). The stem cell types evaluated include bone marrow-derived mesenchymal stem cells (BMSCs), peripheral blood stem cells (PBSCs), umbilical cord mesenchymal stem cells (UCMSCs), adipose-derived stem cells (ADSCs), neural stem cells (NSCs), neural progenitor cells (NPCs), and olfactory ensheathing cells (OECs), Olfactory Mucosa Autografts (OMA) and Olfactory Mucosa Lamina Propria (OM-LP), along with combinatory approaches

Author, year, country	Cell source	Administration route	Number of cells delivered	Time of intervention after injury	Outcomes measure	Functional outcome (%)*; *n* Control group; *n* Treatment group	ASIA grade improvement**
Abdelaziz, O. et al., 2010, Egypt [110]	Autologous BMSCs	Intrathecal and Intralesional injection	5 × 10^6^cells/kg of body weight	6 months – 5 years	ASIA grade	10%; 10; 20	A-C
Deda, H. et al., 2008, Turkey [117]	Autologous BMSCs	Intralesional and Intravenous injection and gel foam covering the spinal cord	Subarachnoid: 5 × 10^6^; Intralesional: 3 × 10^5^; Intravenous: 5 × 10^6^; Gel foam: 1 × 10^7^	2–17 years	ASIA grade	78%; 0; 9	A-C
Geffner, L. et al., 2008, Ecuador [118]	Autologous BMSCs	Intrathecal and Intravenous injection	1.2 × 10^6^ cells/kg	5 days – 21 years	ASIA grade	50%; 0; 8	A-C
Honmou, O. et al., 2021, Japan [119]	Autologous BMSCs	Intravenous injection	0.84–1.6 × 10^8^	1.4 − 1.8 months	ASIA grade	15%/8%; 0; 13	A-C / B-D
Jiang, P-C. et al., 2013, China [120]	Autologous BMSCs	Intrathecal injection or intralesional, computed tomography-guided injection	1 × 10^8^	6 months – >1 year	ASIA grade	5%; 0; 20	A-C
Kakabadze, Z. et al., 2016, Georgia [121]	Autologous BMSCs	Intrathecal injection	7.5 × 10^8^	5 months – 1.67 years	ASIA grade	11%; 0; 18	N.M
Karamouzian, S. et al., 2012, Iran [122]	Autologous BMSCs	Intrathecal injection	7 × 10^5^ to 1.2 × 10^6^	14 days – 1.4 months	ASIA grade	45%; 20; 11	A-C
Kishk, N. et al., 2010, Egypt [123]	Autologous BMSCs	Intrathecal injection	5 × 10^6^ to 10 × 10^6^/kg	3.6 ± 2.5 years	ASIA grade	2%; 20; 43	A-C
Mendonça, M. et al., 2014, Brazil [124]	Autologous BMSCs	Intralesional injection	5 × 10^6^ cells/cm^3^	1.5–15 years	ASIA grade	7%; 0; 14	A-C
Saito, F. et al., 2012, Japan [125]	Autologous BMSCs	Intrathecal injection	3–5 × 10^7^	8–17 days	ASIA grade	20%; 0; 5	B-D
Sharma, A. et al., 2013, India [126]	Autologous BMSCs	Intrathecal and Intramuscular injection	10^6^ cells/kg	< 1 – >5 years	ASIA grade	2%; 0; 110	A-C
Suzuki, Y. et al., 2014, Japan [127]	Autologous BMSCs	Intrathecal injection	2.09–8.44 × 10^8^	24 days – 1 year	ASIA grade	10%; 0; 10	B-D
Syková, E. et al., 2006, Czech Republic [128]	Autologous BMSCs	Transfemoral catheterization of the *arteria vertebralis*	MNCs: 104.0 ± 55.3 × 10^8^; CD34+: 89.7 ± 70.7 × 10^6^	11 days – 1.42 years	ASIA grade	17%; 0; 6	B-D
Vaquero, J. et al., 2016, Spain [129]	Autologous BMSCs	Intrathecal and Intralesional injection	Intrathecal: 30 × 10^6^; Intralesional: 70–200 × 10^6^	3.17–26.75 years	ASIA grade	6%; 0; 17	A-C
Attar, A. et al., 2011, Turkey [130]	Autologous BMSCs	Intralesional injection	MNCs: 19.6-275 × 10^6^ CD34+: 0.96–12 × 10^6^	1 month	ASIA grade	50%; 0; 4	A-C
El-Kheir, W.A. et al., 2014, Egypt [131]	Autologous BMSCs	Intrathecal injection	2 × 10^6^ cells/kg	1–3 years	ASIA grade	4%; 20; 50	A-C
Derakhshanrad, N. et al., 2015, Iran [132]	Autologous BMSCs	Intrathecal injection	4.1 × 10^7^	1.5 months	ASIA grade	100%; 0; 1	A-C
Park, H.C. et al., 2005, South Korea [133]	Autologous BMSCs	Intrathecal and Intralesional injection	98 × 10⁶	7–14 days	ASIA grade	67%; 0; 6	A-C
Al-Zoubi, A. et al., 2014, Jordan [134]	Autologous PBSCs	Intrathecal and Intralesional injection	76 × 10^6^	1–4 years	ASIA grade	11%; 0; 19	A-C
Bryukhovetskiy, A.S., 2015, Russia [108]	Autologous PBSCs	Intrathecal injection	5.8 × 10^6^	< 1 – >5 years	ASIA grade	3%/1%; 20; 202	A-C / A-D
Ichim, T.E. et al., 2010, Costa Rica [109]	Allogeneic UCSCs	Intrathecal and Intravenous injection	UC-MSC: 1 × 10^8^; CD34+: 4.05 × 10^7^	5 months	ASIA grade	100%; 0; 1	A-D
Xiao, Z. et al., 2018, China [135]	Allogeneic UCSCs	Collagen scaffold covering the spinal cord	4 × 10^7^	24 h – 8 days	ASIA grade	100%;0; 2	A-C
Zhu, H. et al., 2016, Hong Kong and Kunmin – China [136]	Allogeneic UCSCs	Intralesional injection	1.6–6.4 × 10^6^	2–20 years	ASIA grade	11%; 0; 28	A-C
Bydon, M. et al., 2020, United States [137]	Autologous ASCs	Intrathecal injection	10 × 10^7^	9 months	ASIA grade	100%; 0; 1	A-C
Bydon, M. et al., 2024, United States [138]	Autologous ASCs	Intrathecal injection	1 × 10^8^	7 months – 1.83 years	ASIA grade	10%; 0; 10	A-C
Ra, J. et al., 2011, South Korea [139]	Autologous ASCs	Intravenous injection	4 × 10^8^	1.07–7.88 years	ASIA grade	13%; 0; 8	A-C
Shin, J. et al., 2015, Republic of Korea [140]	Allogeneic NSCs/NPSCs	Intralesional injection	1 × 10^8^	16 days – 7 months	ASIA grade	11%/5%; 15; 19	A-C / B-D
Huang, H. et al., 2012, China [141]	OECs Autograft	Intralesional injection	Not mentioned	6 months – 30 years	ASIA grade	17%; 0; 108	A-C
Iwatsuki, K. et al., 2016, Japan [142]	Autologous OMA	Implantation	Fill the spinal cord: 1.7–3 cm length	1.4–24.8 years	ASIA grade	50%; 0; 8	A-C
Lima, C. et al., 2006, Portugal [143]	Autologous OMA	Implantation	Fill the spinal cord: 1–4 cm length	6 months – 6.5 years	ASIA grade	29%; 0; 7	A-C
Lima, C. et al., 2010, Portugal [144]	Autologous OMA	Implantation	Fill the spinal cord: 1.3–4 cm length	1.5–15.75 years	ASIA grade	30%; 0; 20	A-C
Larson, C.A. & Dension, P.M., 2013, Portugal and United States [145]	Autologous OMA	Implantation	Fill the spinal cord: 1.3–4 cm length	2.9 ± 1.9 years	ASIA grade	29%; 6; 7	A-C
Rao, Y. et al., 2013, China [146]	Autologous OMA	Subdural injection	48 × 10^6^	8 months – 1.25 years	ASIA grade	25%/12.5%/12.5%; 0; 8	A-C / B-D / C-E
Wang, S. et al., 2016, China [147]	Autologous OM-LP	Implantation	Three to four biopsies of 3 × 3 mm	6 months – 6.92 years	ASIA grade	8%; 4; 8	A-C
Jarocha, D. et al., 2015, Poland [148]	Autologous BMSCs and multiple MSCs	Intrathecal and Intravenous injection	1.54 × 10^8^	2.3 months	ASIA grade	100%; 0; 1	A-C/D
Tabakow, P. et al., 2013, Poland and UK [149]	Autologous OECs and Olfactory Nerve Fibroblasts	Implantation	5 × 10^5^	1.2–5 years	ASIA grade	33%; 3; 3	A-C
Thakkar, U.G. et al., 2016, India [150]	Autologous Adipose Tissue-derived differentiated Neuronal Cells and PBSCs	Intrathecal injection	NSCs (derived from ASCs): 2.4-8 × 10^7^; HSCs: 0.45–1.8 × 10^8^	2.5–5.4 years	ASIA grade	30%/10%; 0; 10	A-C / A-D

* Functional improvement is translated in percentage of patients with ≥2-Grade ASIA improvement from the total of patients treated

** The improvements A–C grade correspond to a change from complete injury to motor incomplete status; B–D correspond to a change from sensory incomplete to stronger motor function; C–E correspond to recovery to normal motor and sensory function; and A–D correspond to recovery from complete injury to motor incomplete with good strength below the injury level

### Traumatic brain injury (TBI)

Up to the date, only a few studies have assessed the safety and efficacy of stem cell approaches for TBI patients. Bone marrow-derived mononuclear cells (BMMNCs) are the most explored in clinical studies, although its use may raise some concerns. BMMNCs comprise a heterogeneous population, including mesenchymal stromal cells, endothelial progenitor cells and a significant part of cells from myeloid, lymphoid and erythroid lineages [151]. Although being an attractive option for cell therapy, the mechanisms behind their therapeutic effect remain poorly understood, due to the presence of diverse cell subtypes with highly distinct roles and interactions [151]. This also poses a barrier for the use of this cell therapy, since cell heterogeneity presents a broader allorecognition, increasing the probability of transplantation rejection of cells from an allogeneic source. Therefore, the selection of this cell source over MSCs may be driven by technical feasibility, rather than therapeutic efficacy and for this reason we have analyzed the effects of the characterized Mesenchymal stromal cells to allow more consistent comparison between trials [152, 153].

MSCs represent an ethical, low immunogenic and easy access source, allowing both allogeneic and autologous transplantation [154]. We selected completed and published clinical studies for TBI, investigating the effect of MSCs either from autologous or allogeneic sources in neurological functions (Table 3). Autologous BMSCs were the most implemented [155, 156], while other studies used allogeneic UCMSCs [157] and modified BMSCs, overexpressing the human plasmid Notch-1 (lowers the potential differentiation into mesodermal lineages and increases their secretion activity) [158]. Transplantation delivery methods comprised direct intracerebral injection [158], intrathecal injection via lumbar puncture [156] and the combination of intralesional with intravenous infusion [155]. Time between injury and treatment varied widely from 1 month to 10 years, existing inconsistency in targeting subacute and chronic phases of TBI, which highlight the need for further studies [159]. Additionally, a 6-month follow-up period was the most employed, except for one study that assessed treatment outcomes at 14 days after transplantation [156]. Unlike standardized outcome measures used in SCI, distinct categories for the follow-up periods post-treatment are present in TBI studies. These reside in clinical measurements of neurological and motor function assessments using different types of measures and scales, along with biochemical tests and imaging techniques (MRI, DTI, CT scan) [88]. This variability is inherent to the conscious and motor status of the patient derived from the different types of trauma, and quantifying therapeutic effects is a challenging aspect for clinical research [160]. Therefore, injury progression and its associated pathophysiological events are more complex, being essential to complement different outcome measures to accurately assess patient’s clinical state and therapy response [161, 162].

In general, no adverse effects were reported from MSCs administration. The combination of intralesional delivery and intravenous re-infusion of BMSCs was reported to lead to significant improvements in neurological severity in the Barthel Index (BI), by the third month and further at 6 months after treatment [155]. Another study indicates that, 2 weeks post BMSCs lumbar puncture, ≈ 40% of patients presented gains in responsive consciousness and motor function after treatment, influenced by patient’s age and treatment timing after trauma [156]. Similarly, UCMSCs efficacy was demonstrated by significant enhancements in extremity motor scores, sensation, balance, as well in communication and social cognition at 6 months after infusion, compared to control [157]. Lastly, intralesional injections of modified BMSCs exhibited significantly higher motor gains on the Fugl-Meyer Motor Scale (FMMS) from baseline, compared to control group at 6 months-post treatment [158]. This effect was more pronounced at higher cell concentration, although no significant differences were reported between doses. Noteworthy, is that the authors did not compare the effects of the modified BMSCs with an unmodified cell source, which limits the conclusions regarding the effectiveness of this modification.

The limited number of clinical studies assessing stem cell therapy in TBI reflects the slow and initial translation of this approach over the last two decades. Moreover, most of the studies are non-randomized, recruited a small number of patients and do not have a control group, limiting the assessment of real functional results. Some key aspects such as optimal cell dosage, administration route and timing remain under investigation and require refinement. This field of research is highly influenced by the timing of treatment post-injury, often limited by logistical and feasibility challenges, which impact the results and their interpretation. In fact, this concern is already recognized in the field of SCI.

**Table 3:** Scientific publications reporting clinical studies on mesenchymal stem cells therapies in TBI patients and the reported functional improvements. The MSCs types evaluated include bone marrow-derived mesenchymal stem cells (BMSCs) and umbilical cord mesenchymal stem cells (UCMSCs)

Author, Year, country	Cell Source	Administration route	Number of cells delivered	Time of intervention after injury	Outcome measures	Functional outcomes (%); *n* Control group; *n* Treatment group
Kawabori et al. (NCT02416492) 2021 USA [158]	Allogeneic modified BMSC (SB623 cells)	Intralesional	2.5,5,10 × 10^6^	> 1y	FMMS, DRS, ARAT, GV, T scores of NeuroQOL	260%^1^ 15; 46 (15 for each concentration)
Wang et al. (ChiCTR-TNRC- 11,001,528) 2013, China [157]	Allogenic UCMSC	Lumbar puncture	10 × 10^6^	1-10y	FMA and FIM scores	546%^2^ 20 ; 20
Z.-X. Zhang et al. 2008, China [155]	Autologous BMSC	Intralesional + Intravenous	10^7^-10^9^	Not mentioned	BI	77%^3^ 0; 7
Tian et al. (NCT02028104) 2013 China [156]	Autologous BMSC	Lumbar puncture	5 × 10^6^	1–3 months	PVS and MFE	≈ 40%^4#^ 0; 97

1,2 - These clinical studies have control groups (with no treatment), and these results represent a comparison to control values following 6 months of treatment

3,4 - These clinical studies do not present a control group (with no treatment), and the percentage of functional results was obtained by comparison to baseline values

#This clinical study only assessed the percentage of patients that improved in the mentioned outcome measures. This result represents the % average obtained in the used outcome measures

ARAT- Action Research Arm Test; BMSCs-Bone Marrow Mesenchymal Stem cells; BI- Barthel index; DRS- Disability Rating Scale; FIM- Functional Independence Measure; FMA-Fugl-Meyer Assessment; FMMS-Fugl-Meyer Motor Scale; GV-gait velocity; Neuro-QOL-Neurology Quality of Life questionnaire; MFE- Motor function evaluation; PVS-persistent vegetative state; UCMSCs- Umbilical Cord Mesenchymal Stem Cells

## Secretome and extracellular vesicle based strategies

Although not yet implemented in clinical settings, cell transplantion-free secretome approaches have become well conceptualized, emerging as an alternative to surpass transplantation challenges related to low survival rate and potential risk of tumorigenicity [163]. The concept of secretome refers to the bioactive content resulting from cell’s paracrine activity, which contains a diverse set of soluble and vesicular components such as growth factors, cytokines, enzymes, bioactive lipids, metabolites and regulatory microRNAs. Together, they have shown neuroprotective, angiogenic, anti-apoptotic and anti-inflammatory properties [99, 164]. Noteworthy, this approach has been proven to have similar regenerative effects to transplantation, since the reported therapeutic relevance of MSCs is mostly derived from their secretory activity [165–167]. These bioactive factors offer a multifaceted and minimally invasive strategy to improve functional recovery.

In preclinical settings, protocols for secretome collection for injections are well established. Typically, cells are expanded to the desired passage, harvested, counted, and reseeded in appropriate growth medium. After appropriate cell expansion, cells are washed and incubated in fresh medium with antibiotics as needed. The conditioned medium termed (secretome) is then collected, snap-frozen in liquid nitrogen, and stored at − 80 °C until use [168]. This approach allows for standardized preparation of bioactive secretome while preserving its therapeutic properties. Importantly, a proteomic analysis comparing the secretome of the most employed MSCs sources has already been made, allowing the identification of the molecules behind its protective and regenerative actions [169, 170].

### Spinal cord injury (SCI)

BMSC-derived extracellular vesicles (EVs) have been shown to significantly improve hindlimb motor function and reduce pro-apoptotic proteins such as cleaved caspase 3 and BCL2 associated X, apoptosis regulator (Bax). These effects are linked to downregulation of the TLR4 and NF-κB phosphorylation and the inhibition of pro-inflammatory factors secretion [171]. EVs also deliver TIMP2, which inhibits matrix metalloproteinases (MMPs) and prevents junctional breakdown, further stabilizing the Blood-Spinal Cord Barrier (BSCB) [172]. Similarly, BMSC-derived EVs limit pericyte migration and preserve BSCB integrity, which together reduce secondary injury and support motor recovery [173]. Administration of BMSC secretome in SCI rats was reported to restore B-cell lymphoma 2 (BCL-2), oligodendrocyte transcription factor 2 (Olig2), growth-associated protein 43 (GAP-43), heat shock protein 70 (HSP70), and tissue plasminogen activator inhibitor II (TPXII) expression levels after SCI, showing the role of secretome in inducing axonal regeneration, survival, oligodendrogliogenesis and protecting cells against stress and oxidation, translating into locomotor function improvement [174].

The ASCs secretome modulates microglia activity, promoting a shift towards an anti-inflammatory state, characterized by increased circulating levels of IL-4 and IL-13 and reduced TNF-α and IL-6 after SCI. This alteration of the cytokine profile through ASCs secretome application creates a more permissive environment for axonal regeneration and contributes to the reduction of glial scarring [175]. Notably, increasing anti-inflammatory cytokines in the context of SCI can also be achieved through hydrogel-based delivery system [176], providing an alternative or complementary therapeutic strategy.

The effect of the ASC secretome have also been investigated in *Xenopus laevis* tadpoles and in mice [175]. In these studies, functional recovery was associated with axonal sprouting in tadpoles and with white matter preservation in mice. Furthermore, the observed functional improvements correlated with decreased levels of pro-inflammatory factors such as IL-1β, IL-6, IFN-γ along with increased levels of IL-4 expression [175]. Beyond immunomodulatory effects, the ASCs secretome is constitutively enriched in ECM proteins such as thrombospondin-1, periostin and collagen fragments. These extracellular matrix (ECM) proteins support angiogenesis, control the inflammation and also promote structural repair, after injury. It also contains vessel-stabilizing factors, including olfactomedin-like 3 as well neurotrophic proteins, all of which synergistically contribute to spinal cord regeneration [177]. Comparing to each fraction alone, the full ASC secretome is shown to be more effective, consistent with a synergistic protein–vesicle mechanism in SCI [178]. The regenerative processes promoted by ASCs secretome involve pathways such as Vascular Endothelial Growth Factor (VEGF), Cadherin and PI3K signaling, which are closely associated with the neurite outgrowth and neuroprotection [87].

Intravenous administration of UCMSC-derived EVs promoted functional recovery and tissue repair in rodent models of spinal cord injury. In rats, EVs reduced neuronal apoptosis by increasing BCL2, decreased Bax, and reduced cleaved caspase 9, while also lowering GFAP and IBA1 expression, indicating a less inflammatory environment [179]. In mice, EVs reduced lesion cavity volume, shifted macrophage populations toward reparative phenotypes, decreased pro-inflammatory cytokines (TNF-α, IL-6, IFN-γ, Granulocyte Colony-Stimulating Factor (G-CSF), Monocyte Chemoattractant Protein-1 (MCP-1) and Macrophage Inflammatory Protein-1 alpha (MIP-1α) [180]. UCMSC-derived EVs inhibited phosphorylation of P38, JNK, ERK, and P65 in microglia and SCI tissue, acting in the context of inflammation, stress response, cell survival, and apoptosis [181].

Importantly, these protective molecules represent potential therapeutic targets for SCI interventions. Growth factors and other neurotrophic proteins could be directly modulated or delivered to promote robust propriospinal axon regrowth across lesions, formation of synaptic contacts, and restoration of functional conduction, an approach already explored in preclinical models [182]. Similarly, modulation of inflammation through macrophage polarization [183] and targeting pro-inflammatory cytokines [184] aligns with strategies proposed to improve outcomes by controlling the immune response. In addition, these molecules can be upregulated in stem cell preparations using overexpression techniques such as plasmid or viral transfection [185–189]. These strategies have been shown to potentiate functional recovery in animal models of SCI and TBI. Of note, the neurotrophic factor BDNF has been recently proposed to serve as a biomarker of functional activity of stem cell therapy in SCI [190].

In summary, stem cell-derived full secretome and its vesicular compartment promote spinal cord repair by enhancing axonal regeneration, supporting cell survival, and stabilizing the blood-spinal cord barrier. They also modulate inflammation by suppressing pro-apoptotic and pro-inflammatory pathways and shifting immune cells toward reparative phenotypes. Together, these effects create a permissive environment for tissue repair and functional recovery, highlighting that the molecules identified in EVs and secretome are not only mechanistic mediators of repair but also actionable targets for therapeutic development.

### Traumatic brain injury (TBI)

Pre-clinical studies in rodents have revealed overall improvements both in motor and cognitive functions from MSCs-secretome application, correlated with reduced contusion volume and neuroinflammation, parallel with increased neurogenesis [88]. Specifically, administration of ASCs secretome in a rat model of mild TBI induced a significant reduction in cortical damage and hippocampal cell death, which was correlated to the presence of long noncoding RNAs in this conditioned medium (CM) [191]. The use of UCMSCs CM engrafted in 3D scaffolds lead to nerve fiber regeneration, correlated with injury size decrease, higher levels of angiogenesis and amelioration of microglial reactivity [192]. The same study unveiled an induction of axonal growth markers such as GAP-43 in neural stem cells, reinforcing the potential effect of MSC secretome in promoting regeneration and plasticity [192]. It has been reported that MSCs-exosome intravenous administration has increased the number of neuroblasts, as well as inducing neuron differentiation and migration, leading to reduced neurological deficits [193]. Correspondingly, the up-regulation of several pro-neurogenesis proteins was found in TBI rat model treated with pre-conditioned UCMSCs CM, such as phosphorylated p-β-catenin, Pox1, Neurog2, and NeuroD1 [194].

All histological and molecular findings of MSCs secretome therapeutic effect have been consistently translated into significant behavioural recovery in pre-clinical TBI models, reinforcing its potential benefits for tissue regeneration and neuroplasticity.

## Risks and limitations of cell therapy

In general, stem cell therapies for CNS trauma have demonstrated a favorable safety profile in clinical settings, with minimal adverse effects reported. Nevertheless, several limitations remain associated with this approach, including major challenges related to limited graft survival, bioavailability and technical reproducibility.

Potential risks such as immune rejection and tumorigenicity may also arise, although these appears to be comparatively lower when MSCs are used, relatively to other stem cell sources [195, 196]. MSCs are known to present low immunogenic potential due to their limited expression of MHC I molecules, but the use of allogeneic sources may still elicit an immunological response [197]. Moreover, cellular heterogeneity also imposes a challenge in obtaining consistent therapeutic outcomes, as it is influenced by donor variability, harvesting procedures, and culture conditions [198]. It is important to note that clinical translation of stem cell therapies and secretome-based approaches requires the implementation of Good Manufacturing Practices for a scale-up production and rigorous quality control, which can present significant technical and cost-related limitations [199, 200].

Therapeutic efficacy may also be influenced by age and pathological history of patients [201]. Younger individuals typically have better regenerative capacities due to higher cellular plasticity and are less prone to suffer from age-related diseases, making them favorable candidates for stem cell treatment assessment [202, 203]. They typically present better prognosis following traumatic injuries, being more likely to achieve better outcomes. Chronic conditions such as metabolic disorders and cardiovascular diseases can impair wound healing and disrupt vascular function, negatively affecting the surviving stem cells transplants [204, 205]. These conditions may especially create a hostile milieu for therapeutic mechanisms that mostly rely on cell differentiation and replacement in CNS, such as neural stem cells, due to their intrinsic complex nature vulnerable to environmental changes [206, 207].

## Conclusion

Collectively, cell-based therapies, including diverse stem cell types and their secreted derivatives, promote spinal cord regeneration and enhance neural plasticity by supporting axonal growth, modulating inflammation, and creating a permissive environment for repair. Clinical evidence demonstrates functional improvements, particularly with BM and PBMSCs, though outcomes vary and further studies are needed to refine strategies. These findings highlight the potential of multifaceted, regenerative approaches to restore structure and function after spinal cord injury.

Although clinical studies of stem cell therapy for TBI are scarce and highly heterogeneous, MSCs sources have been consistently shown to offer distinct regenerative and neuroprotective benefits, supported by behavioral and histological pre-clinical findings. All the reported therapeutic properties converge to the targeting of neuroplasticity and synaptogenesis, while reducing tissue damage and scarring [208, 209].

Overall, stem cell therapies have been shown to support neurogenesis and remyelination processes, ultimately fine-tuning neural circuit connectivity. These processes are critical for restoring neurological function and explain the improvements observed. Nevertheless, translating stem cell therapies into clinical practice still requires further progress. Rigorous and controlled clinical trials are needed, and standardized protocols for administration, dose optimization, and timing must also be established.

## Data Availability

Not applicable.

## Abbreviations

ASCs: Adipose-Derived Stem Cells
ASIA: American Spinal Injury Association
Akt: Protein Kinase B
Bax: BCL2 associated X, apoptosis regulator
BBB: Basso, Beattie, and Bresnahan test
BCL-2: B-cell lymphoma 2
BDNF: Brain Derived Neurotrophic Factor
BI: Barthel Index
BMMNCs: bone marrow-derived mononuclear cells
BMS: Basso mouse scale
BMSCs: Bone Marrow–Derived Mesenchymal Stem Cells
BSCB: Blood-Spinal Cord Barrier
CM: Conditioned medium
CNS: Central Nervous System
CNTF: Ciliary Neurotrophic Factor
CT: Computed Tomography
CSPGs: Chondroitin sulfate proteoglycans
DTI: Diffusion Tensor Imaging
ECM: Extracellular Matrix
ERK: Extracellular Signal-Regulated Kinase
EVs: Extracellular vesicles
FMMS: Fugl-Meyer Motor
GAP-43: Growth-associated protein 43
GCS: Glasgow Coma Scale
G-CSF: Granulocyte Colony-Stimulating Factor
GDNF: Glial cell line-Derived Neurotrophic Factor
GFAP: Glial Fibrillary Acidic Protein
HSCs: Hematopoietic Stem Cells
HSP70: Heat shock protein 70
HSPA1b: Heat Shock Protein Family A Member 1B~
IFN-γ: Interferon-gamma
IGF-1: Insulin-Like Growth Factor 1
ISNCSCI: International Standards for Neurological Classification of Spinal Cord Injury
IL-13: Interleukin-13
IL-1β: Interleukin-1 beta
IL-4: Interleukin-4
IL-6: Interleukin-6
IL-7: Interleukin-7
JAK: Janus Kinase
JNK: c-Jun N-terminal Kinase
MAG: Myelin-associated glycoprotein
MCP-1: Monocyte Chemoattractant Protein-1
MIP-1α: Macrophage Inflammatory Protein-1 alpha
MEK-1: Mitogen-activated protein kinase-1
MMPs: Matrix Metalloproteinases
MRI: Magnetic Resonance Imaging
MSCs: Mesenchymal Stem Cells
mTor: mechanistic Target of Rapamycin
MPS: Methylprednisolone Sodium Succinate
MyD88: Myeloid Differentiation Primary Response 88
NeuroD1: Neurogenic differentiation factor 1
Neurog2: Neurogenin 2
NF-κB: Nuclear Factor kappa-light-chain-enhancer of activated B cells
NGF: Nerve Growth Factor
Notch-1: Neurogenic locus notch homolog protein 1
NPCs: Neural Progenitor Cells
NSCs: Neural Stem Cells
NT3: Neurotrophin-3
NTSCI: Non-Traumatic Spinal Cord Injury
OECs: Olfactory Ensheathing Cells
Olig2: Oligodendrocyte transcription factor 2
OMA: Ofatory Mucosal Autograft
PBMCs: Peripheral-blood mononuclear cells
PI3K: Phosphatidylinositol 3-kinase
p38 MAPK: p38 Mitogen-Activated Protein Kinase
PTEN: Phosphatase and Tensin homolog
PDGFR: Platelet-Derived Growth Factor Receptor
Pox1: Acyl-coenzyme A oxidase 1
ROCK: Rho- associated kinase
ROS: Reactive Oxygen Species
SCI: Spinal Cord Injury
STAT3: Signal Transducer and Activator of Transcription 3
TBI: Traumatic Brain Injury
TIMP2: Tissue Inhibitor of metalloproteinase 2
TIMP3: Tissue Inhibitor of metalloproteinase 3
TLR4: Toll-Like Receptor 4
TNF-α: Tumor Necrosis Factor-alpha
TPXII: Tissue plasminogen activator inhibitor II
TSCI: Traumatic Spinal Cord Injury
UCMSCs: Umbilical Cord–Derived Mesenchymal Stem Cells
VCAM-1: Vascular cell adhesion protein 1
VEGF: Vascular Endothelial Growth Factor
